# Feasibility and preliminary clinical results of linac-based Stereotactic Body Radiotherapy for spinal metastases using a dedicated contouring and planning system

**DOI:** 10.1186/s13014-019-1379-9

**Published:** 2019-10-26

**Authors:** Niccolò Giaj-Levra, Maximilian Niyazi, Vanessa Figlia, Giuseppe Napoli, Rosario Mazzola, Luca Nicosia, Stefanie Corradini, Ruggero Ruggieri, Giuseppe Minniti, Filippo Alongi

**Affiliations:** 1 Advanced Radiation Oncology Department, IRCCS Ospedale Sacro Cuore Don Calabria, Negrar di Valpolicella, 37024 Verona, Italy; 2 Department of Radiation Oncology, University Hospital, LMU, Munich, Germany; 3 German Cancer Consortium (DKTK), Munich, Germany; 4 Radiation Oncology Unit, UPMC Hillman Cancer Center|, San Pietro Hospital FBF, Rome, Italy; 5 Università di Brescia, Brescia, Italy

**Keywords:** Spinal, Metastases, Software, Stereotactic, Radiotherapy

## Abstract

**Background:**

Stereotactic radiosurgery (SRS) and stereotactic body radiotherapy (SBRT) are well established local treatment approaches in several cancer settings. Although SBRT is still under investigation for spinal metastases, promising results in terms of a high effectiveness and optimal tolerability have been recently published on this topic. For spinal SBRT, one of the most relevant issues is represented by the inter-observer variability in target definition. Recently, several technological innovations, including specific tools such as multimodality-imaging (computed tomography (CT), magnetic resonance imaging (MRI) and positron emission tomography (PET-CT), automated volumes contouring and planning, could allow clinicians to minimize the uncertainties related to spinal SBRT workflow. Aim of this study is to report the feasibility of the clinical application of a dedicated software (Element®, Brainlab™ Germany) for spinal metastases SBRT.

**Material and method:**

The patient selection criteria for SBRT in spinal metastases were the following: age > 18 years, diagnosis of spinal metastases (*n* ≤ 3), life expectancy > 3 months, controlled primary tumor or synchronous diagnosis and Spinal Instability Neoplastic Score (SINS) ≤ 12 points. All radiation target volumes were defined and planned with the support of the dedicated software Elements® (Brainlab™ Germany). Different dose prescription have been used: 12 Gy in single fraction, 12 Gy, 18 Gy, 21 Gy and 24 Gy in 3 fractions. Toxicity was assessed according to the Common Terminology Criteria for Adverse Events (CTCAE) v4.0. SPSS version 20 was used for statistical analysis.

**Results:**

From April 2018 to April 2019, 54 spinal metastases in 32 recruited patients were treated with Linac-based SBRT. With a median follow-up of 6 months (range 3–12), local control rates at 6 months and 9 months were 86 and 86%, respectively. No adverse events ≥3 grade were observed.

**Conclusions:**

This preliminary experience shows that with respect to acute toxicity and early clinical response, linac-based using Elements® Spine SRS is a feasible and effective approach.

## Introduction

Approximately one third of cancer patients will develop spinal metastases [1]. Spinal metastases can be associated with back pain, neurological symptoms and deterioration in performance status. Historically, surgical resection and conventional palliative radiotherapy, typically 30 Gy in 10 fractions, have been considered the main treatment options for spinal metastases. Nevertheless, these therapeutic options were associated with a limited local control [2] and pain control probability [3].

Several randomized controlled trials [3, 4] and meta-analyses [5] have investigated the use of different hypofractionated treatment schedules in patients with bone metastases. The studies reported that hypofractionated therapies with 1–5 fractions are comparable to conventional palliative fractionation regimens, in terms of complete and overall pain control. The choice of the appropriate fractionation schedule may be made based on other factors, including the anatomical site or performance status and patient access to the hospital [6]. In general, high single dose is usually offered to patients with a short life expectancy or poor performance status [7].

In the last years, a limited advanced state of metastatic disease was recognized. This phase is defined as oligometastatic and it is generally characterized by a limited tumor burden of disease and potentially amenable to local approaches [8, 9].

Significant development in radiological diagnostic tools and new oncological treatments are allowing to improve life expectancy in metastatic patients. Specifically, the prescription of ablative local treatments can potentially influence clinical outcomes in oligometastatic patients [10]. For this reason, a longer survival of metastatic patients supported the possibility to prescribe ablative treatments as an emerging oncological strategy [11].

Stereotactic radiosurgery (SRS) and stereotactic body radiotherapy (SBRT) have been offered in clinical practice for the management of brain metastases [12] and extracranial anatomical site disease [13]. SBRT allows the prescription of high total dose delivered in one or few sessions to small target volumes, minimizing the dose exposure of normal tissue. Two important radiobiological factors justify the use of SBRT in clinical practice: extreme dose prescription induces tumour cell killing through a direct tumoricidal effect and promotes immune-system activation [14]. Additionally, significant technological improvements (such as volumetric modulated arc therapy (VMAT), helicoidal tomotherapy, and robotic accelerators) allowed a dose painting to the target with a steep dose gradient, while image-guided radiotherapy (IGRT), through a daily verification of patient setup, supported a sparing of normal tissues [15].

The use of SBRT in spinal metastases is an innovative therapeutic approach and has been explored in different studies [16, 17], and clinical trials are on-going (e.g. RTOG 0631).

Recently a dedicated software was released for clinical use in target definition and radiation planning for the stereotactic treatment of spinal metastases with Elements™ Spine SRS (Brainlab®, Munich, Germany).

Aim of the current study is to evaluate the feasibility and to report preliminary results in terms of local control (LC) with the use of this innovative method for the spinal SBRT treatment.

## Material and methods

### Patients and treatment

In the current study, we analyzed the target definition and feasibility of a dedicated contouring and planning system for the treatment of spinal metastases. It is a retrospective study. More specifically, patients were consecutively enrolled, clinical data were prospectively collected, whereas data were retrospectively analyzed for the intent of the analysis. Inclusion criteria for SBRT in spinal metastases were as followed: (a) age > 18 years, (b) diagnosis of spinal metastases (*n* ≤ 3), (c) life expectancy > 3 months, (d) controlled primary tumor or synchronous diagnosis, (e) Spinal Instability Neoplastic Score (SINS) ≤ 12 points [18].

Patients underwent CT simulation without contrast media (1-mm slice thickness) for RT planning with a thermoplastic brain or abdominal mask, according to the site of disease. The dedicated software Elements®Spine SRS (BrainLab™, Munich, Germany) was used to co-register the volumetric magnetic resonance imaging (MRI) T1 sequences (rigid plus deformable) or positron emission tomography computed tomography (PET-CT) for the identification of target volume. Due to the absence of reference standard values for maximum standardized uptake value and other semi-quantitative values, we considered PET-CT as positive only on the basis of qualitative visual assessment performed by two experienced nuclear medicine physicians. Specifically, PET-CT was defined as positive if the metabolic activity of fluorodeoxyglucose (FDG) in the lesion was moderately or markedly increased relative to comparable normal structures or surrounding soft tissues. A lesion with no or faint uptake (less than the surrounding tissues of FDG was defined as negative even if a recurrent tumor had been suspected on CT or MRI. The combination of PET-CT and CT images allowed to merge hypermetabolic areas and bone pathological alterations (i.e. sclerotic, osteolytic or mixed) in order to define the target volume. T2 sequences was used for a precise definition of spinal cord and/or cauda equina (rigid only) to the CT simulation. The software using a dedicated anatomical atlas performed segmentation of the organ at risks (OARs) automatically. The following organ at risks (OARs) were delineated: spinal cord, spinal canal, lungs, esophagus, kidneys and cauda equina. The gross tumor volume (GTV) was defined as macroscopic contrast-enhancing lesion on T1-MRI or pathological uptake on PET-CT. In case of PET FDG uptake, a qualitative method, by two different Nuclear Medicine physicians, were adopted. No semi-quantitative parameters were utilized. Of note, in our population of study the FDG uptake was referred to morphologic CT alterations, as previously reported. The clinical target volume (CTV) was created by an expansion of the GTV according to international guidelines [19]. International consensus established that the CTV should include abnormal marrow alterations and an adjacent normal bony in order to avoid a potential subclinical tumor spread in the marrow space. The CTV was cropped towards the spinal canal and the Planning target volume (PTV) was obtained by adding an isotropic margin of 2 mm to the CTV. According to our clinical protocols, the prescribed dose and fractionation were chosen based on the tumor volume, previous spinal radiation treatment and OARs tolerance limits. Corticosteroid therapy was prescribed only if patient reported pain or any neurological symptoms.

### Treatment planning and dosimetric constraints

**Fig. 1:**
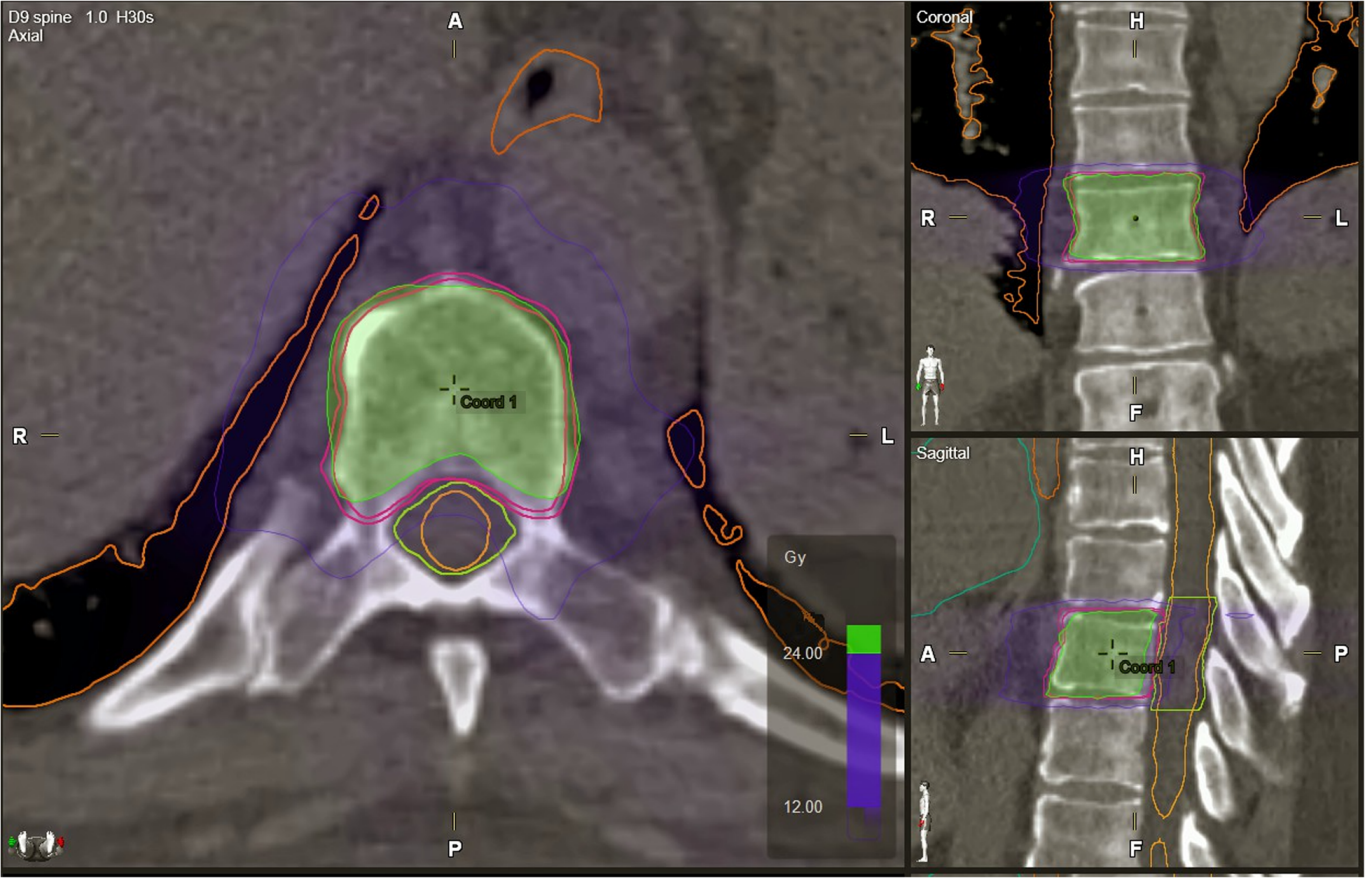
Green represented 100% of dose prescription and blu 50% of dose prescription (24 Gy in 3 fractions)

Treatments were performed with a TrueBeam™ (Varian Medical Systems, Palo Alto, CA, USA) linac, equipped with a Millenium 120-leaves MLC; specifically only central leaves (0.5 cm at isocenter) have been used in the current series. Beam energy was typically 6-10MV flattening-filter-free (FFF). All plans were optimized by Spine-SRS (v. 1.0, Brainlab AG), which is an add-on of Elements™ (Brainlab AG) Treatment Planning Systems, by the use of two 180-degrees consecutive arcs, thus covering a full 360° rotation, but with a distinct collimator rotation at 45° and 315° respectively. Dose distributions were computed, with a 1 mm dose-grid step and 2 degrees of angular step (control point) along the arcs, by a pencil-beam based dose calculation algorithm. In terms of target dose coverage, volumetric dose prescription was 95%D_p_ to 98%PTV, while D_2%_(PTV) as large as 120%D_p_ was accepted when necessary to assure the required sparing of the spinal cord (Fig. 1). A planning organ at risk (PRV) was used for spinal cord with a isotropic margin of 2 mm from true cord. The dose constraints for planning approval according to the sparing of the OARs were as follow: spinal cord 0.1 cc < 14 Gy in single fraction and < 21.9 Gy in three fractions, kidney (200 cc) < 8.4 Gy in single fraction and < 16 Gy in three fractions, esophagus (1 cc, hottest voxel) < 15.4 Gy in single fraction and < 25.2 Gy in 3 fractions, each lung V5 < 35%, V10 < 10%, V20 < 3% and mean dose ≤5 Gy for both fractionations. Radiation treatment was given in consecutive days in all patients. In the treatment room, a cone-beam CT (CBCT) was assessed to verify accuracy of patient set-up.

### Radiological and clinical follow-up

Regarding follow-up, clinical evaluation, PET-CT and/or MRI were performed 45–60 days after SBRT treatment, every 2–3 months during the first year. PET-CT was the principal radiological restaging diagnostic method used to evaluate local response. MRI scan was proposed when unclear response to radiotherapy treatment (e.g. pseudoprogression) was observed or in patients with a suspicious local disease progression. At each visit, neurological status, pain control, and side effects were recorded and scored according to the Visual Analogue Scale (VAS) and Common Terminology Criteria for Adverse Events (CTCAE version 4.0), respectively. Adverse neurological events were considered a consequence of treatment in the absence of progressive disease.

### Statistical analysis

Local control (LC) was defined as a lack of progression in the radiotherapy field of the treated metastatic lesions (i.e., any response or stable disease). This was calculated from the beginning of SBRT to local relapse date. It was estimated by the Kaplan-Meier method. Statistical analyses were carried out using SPSS 20. Clinical outcomes and toxicity data according to the CTCAE version 4.0 and VAS were collected prospectively. According to the limited follow-up, we did not performed any univariate and multivariate analysis.

## Results

From April 2018 to April 2019, 54 spinal metastases in 32 patients were treated with Linac-based SBRT (Table 1). Median age was 68 years (range 43–83 years), male and female were 21 (65.6%) and 11 (34.4%), respectively. Median performance status (PS) was 1 (range 0–2). In 13 patients (42%) the primary diagnosis was prostate cancer, followed by breast cancer in 9 cases (29%). The cervical spine was involved in 7 (13.5%) cases, the thoracic spine in 35 (67.2%), and the lumbar spine in 10 cases (19.3%). In all cases, non-contiguous spinal lesions have been treated. Anatomical parts of the vertebral body affected by the tumor was the vertebral body in 27 metastases (50%), the peduncles was involved in 8 cases (14.8%), spinal process in 4 (7.4%) and mixed vertebral involvement in the residual 15 cases (27.8%). At diagnosis, a SINS value between 0 and 6 (stable vertebra) was documented in 22 cases (68.7%), while an intermediate stability (value between 7 and 12) was reported in 10 (31.3%). Additionally, median pain level (VAS) at first clinical evaluation was 0 (range 0–8). The median number of spinal metastases treated with SBRT was 1 (range 1–3). All patients did not reported any neurological symptoms before radiation treatment. For the radiation target definition, MRI scan was used in 5 cases (15.6%), PET-CT in 22 patients (68.8%) and a both in 5 (15.6%). Regarding systemic therapy, 8 patients (25.8%) were not treated with ongoing concomitant treatment, while regarding the other patients: 15 (48.4%) were treated simultaneously with hormonal therapy, 4 (12.9%) with chemotherapy and 4 (12.9%) with immunotherapies. Patients receiving systemic therapies interrupted the medical treatment a week before stereotactic radiotherapy and resumed seven days by the end of SBRT.

**Table 1:** Patients and disease characteristics

Number of patients and spinal metastases	32 and 54
Sex (F/M)	11/21
Median age (range)	68 (43–83 years)
Median performance status (range)	1 (0–2)
Histology (%)	
Lung	2 (6.2%)
Breast	9 (28.1%)
Prostate	15 (46.9%)
Others	6 (18.8%)
Median number of spinal metastases (range)	1 (1–3)
Pre-treatment MRI	5 (15.6%)
Pre-treatment PET-CT	22 (68.8%)
Pre-treatment combined radiological exams	5 (15.6%)
Vertebra (%)	
Cervical	7 (13.0%)
Dorsal	36 (66.7%)
Lumbar	11 (20.3%)
Sacral	0
Anatomical site (%)	
Vertebral body	27 (50%)
Vertebral body + spinal process	1 (1.9%)
Vertebra body + peduncle	8 (14.8%)
Vertebral body + spinal process + peduncle	4 (7.4%)
Peduncle	8 (14.8%)
Spinal process	4 (7.4%)
Full vertebra	2 (3.7%)
Spinal Instability Neoplastic Score (SINS)	
Median (range)	5
0–6	22 (68.7%)
7–12	10 (31.3%)
Pre-treatment Visual Analogue Scale (VAS)	
Median (range)	0 (range 0–8)
VAS at first follow-up	0 (range 0–7)
VAS at Post-treatment	
Median (range)	0 (range 0–7)
Systemic therapy combined with SBRT (no; %)	
None	8 (25.0%)
Hormone therapy	16 (50%)
Chemotherapy	4 (12.5%)
Target therapy	0
Immunotherapy	4 (12.5%)

Treatment characteristics are summarized in Table 2. Median GTV, CTV and PTV volumes were 3.3 cc (range 1.2–53.8 cc), 20.3 cc (range 5.0–136.1) and 26.5 cc (range 7.6–250.6 cc), respectively.

**Table 2:** Treatment characteristics

Number of patients and spinal metastases	32 and 54
Median GTVcc (range)	3.3 (0.2–53.8)
Median CTVcc (range)	20.3 (5.0–136.1)
Median PTVcc (range)	26.5 (7.6–250.6)
Spinal treatment (%)	
SBRT (single dose)	4 (7.5%)
12 Gy in 1 fractions	4 (100%)
SBRT (multiple doses)	49 (92.5%)
4 Gy in 3 fractions	2 (4.1%)
6 Gy in 3 fractions	13 (26.5%)
7 Gy in 3 fractions	20 (40.8%)
8 Gy in 3 fractions	14 (28.6%)
Dose prescription	
Median (Gy; range)	21 Gy (12–24 Gy)

The majory of the spinal lesions were treated in 3 fractions. Twelve Gy in 1 and 3 fractions were used in 4 patients as re-irradiation (all patients received a previous dose prescription of 30 Gy in 10 fractions), 18 Gy in 3 fractions was used in 13 lesions (26.5%), 21 Gy in 3 fractions in 20 (40.8%), 24 Gy in 3 fractions in 14 spinal metastases (28.6%), a dose prescription of 21 Gy in 3 fractions was prescribed in 20 lesions (36.7%), 24 Gy in 3 fractions in 13 lesions (26.5%). A SBRT approach with a single fraction was offered to 4 lesions and with a median dose prescription of 12 Gy – Table 2. In highly selected cases – 8 cases (i.e. radio-resistant histology and/or well defined very small and focal GTV area) a simultaneous integrated boost to the GTV was offered (range dose 27–30 Gy). With regard to the dosimetric constraints, the software was able to satisfy dosimetric values in terms of tolerability to OARs and only in 5 lesions physic modified manually treatment planning. Median dose constrains are reported in Table 3.

**Table 3:** Dosimetric data to organ at risk and target volumes

Spinal cord (0.1 cc)	
Single fraction (median - range)	7.8 (5.08–10.47)
Three fractions (median - range)	15.5 (7.3–19.5)
Lung	
Single fraction (median - range)	
V5%	0.25 (0.03–0.46)
V10%	0
V20%	0
Mean Dose	1.79 (1.1–8.58)
Three fractions (median – range)	
V5%	4.4 (0.42–24.1)
V10%	0.6 (0.01–5.7)
V20%	0 (0–1.21)
Mean dose	1.11 (0.35–4.91)
Esophagus (1 cc)	
Single fraction (median - range)	6.86 (6.16–7.56)
Three fractions (median - range)	11.3 (0.03–15.4)
Kidney (> 200 cc)	
Single fraction (median - range)	3.4 (0.2–5.1)
Three fractions (median - range)	8.2 (1.2–13.2)

### Local control, toxicity and dosimetry

**Fig. 2:**
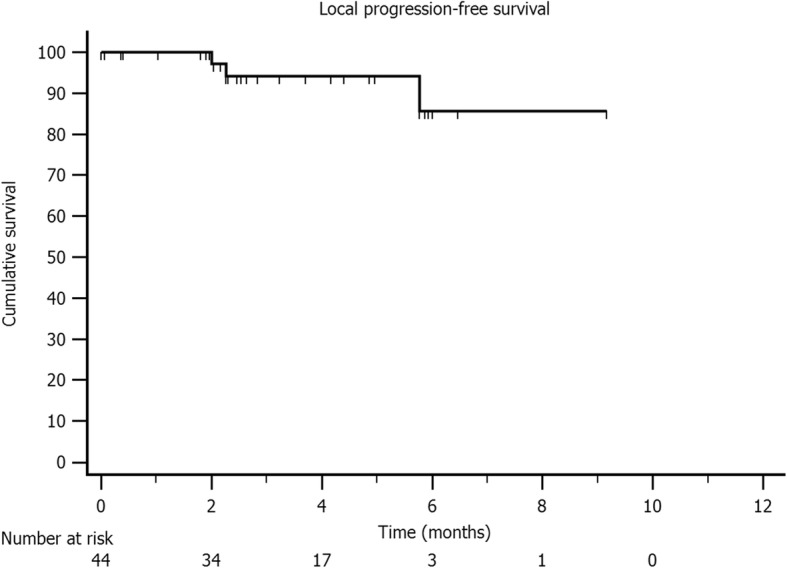
Local control probability after SBRT

At a median follow-up of 6 months (range 3–12 months), local control rates at 6 months and 9 months were 86 and 86%, respectively (Fig. 2).

Only one single patient interrupted the radiation treatment due to a worsening in general clinical condition. Within 24–48 h from the start of the treatment, 2 out of 32 patients reported a pain exacerbation, which was successfully controlled by increasing their doses of anti-inflammatory drugs and/or steroids without interrupting the treatment. At the first follow-up, the median VAS remained 0 (range 0–8) and was also confirmed at the last follow-up. A VAS reduction was observed in 5 patients, with a median reduction of 2 points; while in 3 patients we observed a stable VAS value. No acute or chronic adverse events ≥3 grade were reported at the follow-up. At the time of the analysis, in all patients a vertebral fracture compression was not recorded.

## Discussion

Radiotherapy is considered a highly effective local approach for patients with spinal metastases. The two main reasons for the large clinical use of radiotherapy in this setting are the ability of pain relief and the attempt to achieve cytoreductive effects on local disease to prevent possible neurological deterioration. In this clinical scenario, SBRT is an innovative approach capable of delivering high radiation doses that potentially improve LC rates, as reported in patients with brain and extracranial metastases and early-stage non-small cell lung cancer [20, 21]. Notably, a recent randomized phase II trial demonstrated that the use of SBRT in spinal metastases was associated with a faster and improved pain response compared to conventional fractionated palliative radiotherapy [16].

In the current study, we have reported our initial experience with a dedicated treatment planning software for spinal SBRT. With a median follow-up of 6 months, LC rates were 86 and 86% at 6 and 9 months respectively. In addition, the treatment was well tolerated, with no severe (≥ 3 grade) acute toxicity recorded at the follow-up. Our results are consistent with those reported by others, indicating that SBRT is an effective and safe treatment option for spinal metastases, with a limited risk of complications, including vertebral compression fracture (9.5%), symptomatic myelopathy (0.2%), esophageal toxicity and flair pain [22, 23].

In the last decades, a significant improvement in radiological definition, imaging resolution and metabolic information (MRI and PET-CT) allowed an early detection of spinal metastases, favoring the prescription of ablative radiation treatments. In ablative radiation treatments, target volume outline and definition of organ at risk are crucial steps, in order to reduce the probability of toxicity or target missing. Nevertheless, inter-observer variability in target delineation and organ at risk definition still remains a relevant issue in modern radiotherapy, including spinal SBRT [24]. Two different methods can reduce inter-observer variability: educational tools and atlases [18–24], as well as auto-contouring software. In our clinical experience, the Spine SRS dedicated software has been used and its implementation has been associated to different practical advantages in our clinical workflow. In our experience this software was able to optimize multimodality-imaging fusion (PET-CT and or MRI images) to simulation CT scans in all cases, by a deformable imaging registration to correct postural set-up errors during the positioning during the various exams by focusing on the vertebral column segments. In comparison to a manually performed image alignment, fusion and contouring procedures, a significant time saving for the entire automatic process was recognized by using the software. For this reason, an accurate comparison between manual and automatic software-guided procedures in terms of timing will be the subject of further analyses, focusing more on cost and time effectiveness. In our experience, in all cases, including various challenging situation were the CTV was very close or in contact with the relative OAR, treatment planning constraints were satisfied, as reported in Table 3. Additionally, patients selection was accurate. Inclusion criteria for SBRT in spinal metastases were as followed: (a) age > 18 years, (b) diagnosis of spinal metastases (*n* ≤ 3), (c) life expectancy > 3 months, (d) controlled primary tumor or synchronous diagnosis, (e) Spinal Instability Neoplastic Score (SINS) ≤ 12 points. More specifically, most of them were with small and limited targets without neurological symptoms or pain. This kind of population represents the typical clinical profile of so-called oligometastatic patients. In this last context, recent literature is starting to demonstrate that the combination of ablative treatment and systemic therapies could impact on clinical outcomes [25]. Compared to invasive local therapies for vertebral metastases including radiofrequency ablation, cryosurgery, the prescription of non-invasiveness SBRT, especially when supported by tools able to improve its workflow and its feasibility, represents an appealing feature in the panorama of local treatment options.

## Conclusions

The Elements® Spine SRS dedicated software for linac-based spinal SBRT treatment is a fast and effective approach for patients with spinal metastases; our preliminary experience confirms the feasibility in the clinical work-flow of this innovative approach. Although larger series with longer follow-up are needed to confirm the high local control rates and safety profiles reported in the current study, our preliminary experience clearly suggests that this innovative software for spinal SBRT represents an intriguing and easily adoptable treatment method with potential advantages in daily clinical practice and in treatment planning accuracy.

## Data Availability

The patient information may be shared under ‘IRCCS Sacro cuore – Don Calabria’ hospital IRB approval of amendment on a case by case base. The Element™ planning package is proprietary (Varian Medical Systems, Palo Alto, CA) due to patent protection.

## Abbreviations

CT: Computed tomography
CTCAE: Common Terminology Criteria for Adverse Events
CTV: Clinical target volume
D: Dose
GTV: Gross tumor volume
IGRT: Image-guided radiotherapy
LC: Local control
MRI: Magnetic resonance imaging
OARs: Organ at risks
PET-CT: Positron emission tomography computed tomography
PTV: Planning target volume
SBRT: Stereotactic body radiotherapy
SINS: Spinal Instability Neoplastic Score
V: Volume isodose
VAS: Visual Analogue Scale
VMAT: Volumetric modulated arc therapy

## References

[1] Wong DA, Fornasier VL, MacNab I (1990). Spinal metastases: the obvious, the occult, and the impostors. Spine.

[2] Sakaura H, Hosono N, Mukai Y, Ishii T, Yonenobu K, Yoshikawa H (2004). Outcome of total en bloc spondylectomy for solitary metastasis of the thoracolumbar spine. J Spinal Disord Tech.

[3] Maranzano E, Bellavita R, Rossi R, De Angelis V, Frattegiani A, Bagnoli R (2005). Short-course versus split-course radiotherapy in metastatic spinal cord compression: results of a phase III, randomized, multicenter trial. J Clin Oncol.

[4] Tong D, Gillick L, Hendrickson FR (1982). The palliation of symptomatic osseous metastases: final results of the study by the irradiation therapy oncology group. Cancer.

[5] Chow E, Zeng L, Salvo N, Dennis K, Tsao M, Lutz S (2012). Update on the systematic review of palliative radiotherapy trials for bone metastases. Clin Oncol (R Coll Radiol).

[6] Koswig S, Budach V (1999). Remineralization and pain relief in bone metastases after after different radiotherapy fractions (10 times 3Gy vs. 1 time 8Gy). A prospective study. Strahlenther Onkol.

[7] Silva MF, Marta GN, Lisboa FPC, Watte G, Trippa F, Maranzano E (2019). Hypofractionated radiotherapy for complicated bone metastases in patients with poor performance status: a phase II international trial. Tumori..

[8] Alongi F, Mazzola R, Ricchetti F (2017). Consolidative local therapy in oligometastatic patients. Lancet Oncol.

[9] Ricardi U, Giaj Levra N, Badellino S, Alongi F (2017). Role of consolidative stereotactic ablative radiotherapy in patients with oligometastatic non-small cell lung cancer. J Thorac Dis.

[10] Gomez Daniel R., Tang Chad, Zhang Jianjun, Blumenschein George R., Hernandez Mike, Lee J. Jack, Ye Rong, Palma David A., Louie Alexander V., Camidge D. Ross, Doebele Robert C., Skoulidis Ferdinandos, Gaspar Laurie E., Welsh James W., Gibbons Don L., Karam Jose A., Kavanagh Brian D., Tsao Anne S., Sepesi Boris, Swisher Stephen G., Heymach John V. (2019). Local Consolidative Therapy Vs. Maintenance Therapy or Observation for Patients With Oligometastatic Non–Small-Cell Lung Cancer: Long-Term Results of a Multi-Institutional, Phase II, Randomized Study. Journal of Clinical Oncology.

[11] Massicotte E, Foote M, Reddy R, Sahgal A (2012). Minimal access spine surgery (MASS) for decompression and stabilization performed as an out-patient procedure for metastatic spinal tumours followed by spine stereotactic body radiotherapy (SBRT): first report of technique and preliminary outcomes. Technol Cancer Res Treat.

[12] Alongi F, Fiorentino A, Gregucci F, Corradini S, Giaj-Levra N, Romano L (2019). First experience and clinical results using a new non-coplanar mono-isocenter technique (HyperArc™) for Linac-based VMAT radiosurgery in brain metastases. J Cancer Res Clin Oncol.

[13] Ricardi U, Filippi AR, Franco P (2013). New concepts and insights into the role of radiation therapy in extracranial metastatic disease. Expert Rev Anticancer Ther.

[14] Giaj-Levra N, Sciascia S, Fiorentino A, Fersino S, Mazzola R, Ricchetti F (2016). Radiotherapy in patients with connective tissue diseases. Lancet Oncol.

[15] Sahgal A1, Bilsky M, Chang EL, Ma L, Yamada Y, Rhines LD (2011). Stereotactic body radiotherapy for spinal metastases: current status, with a focus on its application in the postoperative patient. J Neurosurg Spine.

[16] Sprave T, Verma V, Förster R, Schlampp I, Bruckner T, Bostel T (2018). Randomized phase II trial evaluating pain response in patients with spinal metastases following stereotactic body radiotherapy versus three-dimensional conformal radiotherapy. Radiother Oncol.

[17] De Bari B, Alongi F, Mortellaro G, Mazzola R, Schiappacasse L, Guckenberger M (2016). Spinal metastases: is stereotactic body radiation therapy supported by evidences?. Crit Rev Oncol Hematol.

[18] Spratt DE, Beeler WH, de Moraes FY, Rhines LD, Gemmete JJ, Chaudhary N (2017). An integrated multidisciplinary algorithm for the management of spinal metastases: an international spine oncology consortium report. Lancet Oncol.

[19] Cox BW, Spratt DE, Lovelock M, Bilsky MH, Lis E, Ryu S (2012). International spine radiosurgery consortium consensus guidelines for target volume definition in spinal stereotactic radiosurgery. Int J Radiat Oncol Biol Phys.

[20] Gregucci F, Fiorentino A, Corradini S, Figlia V, Mazzola R, Ricchetti F (2019). Linac-based radiosurgery or fractionated stereotactic radiotherapy with flattening filter-free volumetric modulated arc therapy in elderly patients : a mono-institutional experience on 110 brain metastases. Strahlenther Onkol.

[21] Ricardi U, Filippi AR, Guarneri A, Giglioli FR, Ciammella P, Franco P (2010). Stereotactic body radiation therapy for early stage non-small cell lung cancer: results of a prospective trial. Lung Cancer.

[22] Husain ZA, Sahgal A, De Salles A, Funaro M, Glover J, Hayashi M (2017). Stereotactic body radiotherapy for de novo spinal metastases: systematic review. J Neurosurg Spine.

[23] Chiang A, Zeng L, Zhang L, Lochray F, Korol R, Loblaw A (2013). Pain flare is a common adverse event in steroid-naïve patients after spine stereotactic body radiation therapy: a prospective clinical trial. Int J Radiat Oncol Biol Phys.

[24] De Bari B, Dahele M, Palmu M, Kaylor S, Schiappacasse L, Guckenberger M, ESTRO FALCON core (2018). Short interactive workshops reduce variability in contouring treatment volumes for spine stereotactic body radiation therapy: experience with the ESTRO FALCON programme and EduCase™ training tool. Radiother Oncol.

[25] Palma DA, Olson R, Harrow S, Gaede S, Louie AV, Haasbeek C (2019). Stereotactic ablative radiotherapy versus standard of care palliative treatment in patients with oligometastatic cancers (SABR-COMET): a randomised, phase 2, open-label trial. Lancet.

